# Commentary: Fairness is intuitive

**DOI:** 10.3389/fpsyg.2016.00654

**Published:** 2016-05-09

**Authors:** Kristian O. R. Myrseth, Conny E. Wollbrant

**Affiliations:** ^1^School of Management, University of St AndrewsSt Andrews, Scotland; ^2^Department of Economics, School of Business, Economics and Law, University of GothenburgGothenburg, Sweden

**Keywords:** fairness, self-control, intuition, decision times, dictator game

Cappelen et al. (2015) open their paper, “Fairness is intuitive,” with the observation, “A key question in the social sciences is whether it is intuitive to behave in a fair manner or whether fair behavior requires active self-control” (p. 2). They purport to offer “evidence showing that fair behavior is intuitive to most people” (p. 1). Their premise is that deciding by intuition is faster than deciding by deliberation. While this premise in and on itself is rather uncontroversial—the conclusion that they draw from it is not: “Since a decision that relies on intuition is typically made faster than a decision that relies on deliberation, the response time of a fair decision relative to a selfish decision provides an important indication of the intuitiveness of fair behavior” (p. 2). This reasoning, in fact, amounts to a reverse inference fallacy^1^. “Intuitive” may mean “fast,” but this would not imply that “fast” means “intuitive.”

However, we may ask, under which empirical conditions might we be allowed to draw the inference of “intuitive” from “fast”? Naturally, these conditions would require that “fast” rule out “deliberative.” To achieve this, we would need information beyond relative response speed alone—such as absolute decision times. And this begs the question, which range of decision times would rule out “deliberative”—or at the very least, render it improbable? Although the precise cut-off for deliberative decisions may be difficult to establish (see e.g., Schneider and Shiffrin, 1977; Posner and Rothbart, 1998), it is clear that an individual, if given a few seconds, may have sufficient time to reflect consciously—and ample time, if given more than thirty. Responses made at those speeds ought thus not be taken as “intuitive” prima facie, on the basis of the response time data alone. Unfortunately, the authors make just this mistake.

Cappelen et al. (2015) find that “fair” decisions in a dictator game are faster than are “selfish” decisions, from which they infer that the fair decision is the more intuitive (e.g., Figure 2, p. 4). However, fair decisions took on average 38.4 s, and unfair decisions on average 48.5. It would seem, then, that both decision categories are fairly slow—and neither would appear unlikely to be characterized by deliberative processes. We may speculate about sources of the difference in mean response times, but intuitive as opposed to deliberative decision making is but one out of multiple possible explanations. Another explanation, for example, could be differences in degrees of deliberation. That is, individuals who deliberated more extensively might have reached a selfish decision, whereas individuals who deliberated less—but who did deliberate nonetheless—might have arrived at a fair choice. It is even possible, in this scenario, that the impulsive response is selfish—as some prior literature has suggested (e.g., Martinsson et al., 2012; Achtziger et al., 2015). The spontaneous response may then have been overruled by controlled deliberation, which might have been overturned yet again by even more extensive deliberation. In other words, individuals might have experienced an initial proclivity, changed their mind, and then changed their mind once again. As this possible scenario shows, it would be very difficult, to assign “fair” as opposed to “selfish” responses to intuition over deliberation.

Although Cappelen et al. (2015) make the valuable point of distinguishing conceptually between actual decision time and overall measured response time—which encompasses also reading time and decision implementation time—their distinction does not salvage their conclusion. Indeed, their measured response times include the time spent on reading and comprehending the instructions, but any such activity—by its very nature—would require some degree of deliberation. Therefore, it would not be possible subsequently—on the basis of relative response times alone—to distinguish between intuitive and deliberative decision processes^2^. A very fast decision, for example, may be the product of deliberation during the preceding reading and comprehension steps.

Cappelen et al. (2015) build on the work by Rand et al. (2012, 2014), who fall into similar traps. Rand et al. (2012, 2014) argue that time-pressure promotes “cooperation,” and that this amounts to evidence for the notion that cooperation is intuitive^3^. However, subjects in their time-pressure treatments had adequate time to deliberate—median response times were 6–13 s, across studies. As Myrseth and Wollbrant (2015) argue, this calls into question the meaning of the time-pressure treatments. Although Rand et al. (2012, 2014) also show that cooperation is negatively associated with response time, a closer examination of their data, in which average cooperation rates are plotted against response times, reveals that the pattern is non-linear and generally unclear (Myrseth and Wollbrant, 2015). In fact, when examined locally, there appears to be a positive association between response times and cooperation, among decisions made within 4 s^4^. A negative pattern emerges for slower decisions. The data from Rand et al. (2012, 2014) thus fail to provide meaningful evidence for the hypothesis that cooperation is intuitive rather than deliberative.

More generally, we would call for greater caution in the interpretation of response time data. Although often fast, intuition can also be slow, and, conversely for deliberation—although often slow, it can also be fast (within limits). It is therefore not straightforward to rely on response times—or on experimental time pressure treatments—to disentangle intuition from deliberation in economic decision making.

## Author contributions

All authors listed, have made substantial, direct, and intellectual contribution to the work, and approved it for publication.

### Conflict of interest statement

The authors declare that the research was conducted in the absence of any commercial or financial relationships that could be construed as a potential conflict of interest.

## Footnotes

^1^Another term for “reverse inference fallacy,” is the “fallacy of affirming the consequent”, as defined by Dowden (2016).

^2^A reviewer pointed out that our argument could be construed as a blanket dismissal of the utility of response times as a process measure. We would stress, however, that our argument applies to empirical and theoretical contexts similar to that of the target paper, and we recognize that response time measurement has its uses. Examples of insightful application of response times in economic decision-making include Rubinstein (2007) and Achtziger and Alós-Ferrer (2014).

^3^As Cappelen et al. (2015) note, the empirical stability of the pattern obtained by Rand et al. (2012, 2014) is contested. Tinghög et al. (2013) and Verkoeijen and Bouwmeester (2014) fail to reproduce the pattern. Moreover, Tinghög et al. (2013) and Recalde et al. (2015) argue that the original pattern may have arisen from analytical and methodological artifacts, respectively.

^4^This discussion refers to an analysis that pools the data from all studies in Rand et al. (2014). The pattern is largely consistent when only one-shot public good games are considered. For further details, see Myrseth and Wollbrant (2015).
